# Connectivity Reveals the Relationships between Human Brain Areas Associated with High-Level Linguistic Processing and Macaque Brain Areas

**DOI:** 10.3390/tomography10070082

**Published:** 2024-07-12

**Authors:** Fangyuan Wang, Xiaohua Lu, Xiaofeng Chen, Qianshan Wang, Qi Li, Haifang Li

**Affiliations:** 1 School of Computer Information Engineering, Shanxi Technology and Business University, Taiyuan 030024, China; wangfangyuan@sxtbu.edu.cn (F.W.); luxiaohua@sxtbu.edu.cn (X.L.); 2 College of Computer Science and Technology, Taiyuan University of Technology, Taiyuan 030024, China; 19834007433@163.com (X.C.); wangqianshan@tyut.edu.cn (Q.W.); liqi0083@link.tyut.edu.cn (Q.L.)

**Keywords:** cross-species comparison, human language region, connectivity, connectivity fingerprint, diffusion MRI

## Abstract

Cross-species research has advanced human understanding of brain regions, with cross-species comparisons using magnetic resonance imaging technology becoming increasingly common. Currently, cross-species research on human language regions has primarily focused on traditional brain areas such as the Broca region. While some studies have indicated that human language function also involves other language regions, the corresponding relationships between these brain regions in humans and macaques remain unclear. This study calculated the strength of the connections between the high-level language processing regions in human and macaque brains, identified homologous target areas based on the structural connections of white-matter fiber bundles, and compared the connectivity profiles of both species. The results of the experiment demonstrated that macaques possess brain regions which exhibit connectivity patterns resembling those found in human high-level language processing regions. This discovery suggests that while the function of a human brain region is specialized, it still maintains a structural connectivity similar to that seen in macaques.

## 1. Introduction

Language is a fundamental cognitive skill that is uniquely and universally human. Understanding language involves studying the brain, particularly the brain regions responsible for language processing. Clinical research supports the study of early language regions, with Broca’s area in the posterior part of the inferior frontal gyrus and the Wernicke area in the superior temporal gyrus being identified as key language-function areas [1]. Broca’s area is associated with language production, while Wernicke is involved in language reception and comprehension, leading to the development of the classical Wernicke–Lichtheim–Geschwind language model [2]. Further research using cortical electrical stimulation has revealed that the language center comprises multiple brain regions, indicating that language processing involves a network of regions [3]. Functional connectivity studies have shown that other language-related brain regions are predominantly located in the left temporal lobe, left inferior frontal gyrus, and left middle frontal gyrus, as well as areas extending into the angular gyrus [4,5]. Researchers are not only interested in identifying the brain regions involved in language processing but also studying the characteristics of these regions, including language mechanisms, lateralization, and cross-language features. While human language is considered more complex and versatile than nonhuman communication systems, studies have shown that nonhuman primates, such as apes and monkeys, exhibit some linguistic properties similar to those in human language, highlighting the potential importance of studying nonhuman primates in understanding human language.

Comparative neurobiology is a commonly used method in brain research. Comparing species provides insight into the evolutionary process of the human brain and advances human understanding of the human brain [6]. Macaque monkeys and chimpanzees have long been considered classic translational models for understanding human brain functions [7], especially cognitive abilities and language [8,9]. An increasing number of studies have focused on the brain regions of nonhuman primates to reveal internal relationships between species in language regions, primarily focusing on frontal, parietal, and temporal brain regions, due to their active response during language comprehension and production in humans. These studies have mainly utilized four methods: electrical stimulation analysis, cell construction analysis, anatomical analysis, and connectivity analysis. Several experiments have shown the presence of regions in the macaque brain similar to the human angular gyrus (AG) [10,11], which is crucial for semantic comprehension and located in the posterior part of the inferior parietal lobule. The homologies of these characteristics have been further emphasized from various perspectives [12]. Studies have also demonstrated that key neuroanatomical features of the ventrolateral frontal cortex (vlFC) in humans seem to be homologous in macaques, with regions in the macaque vlFC showing a functional coupling pattern similar to that found in human vlFC regions involved in language [13]. The classical language area, Broca’s area, is situated in the left vlFC [14], and homologs of Broca’s area in nonhuman primates have been identified through various methods [15,16,17]. Similarly, in nonhuman primates, Tpt is considered a possible homolog of the planum temporale in humans, based on its anatomical connectivity and cytoarchitectonic organization. Human PTs share similar anatomical, positional, and connectional characteristics with chimpanzee PTs. Moreover, studies in nonhominoid primates support the homology of this region [18]. Several nonhuman primate species also exhibit human-like brain asymmetries in areas homologous to key language areas [19,20,21]. Furthermore, although language function in humans involves other brain regions, existing research lacks cross-species comparisons sufficient to determine if brain regions specialized in language processing in humans have similar structures in the monkey brain. This study aims to comprehensively establish cross-species cognition relative to brain regions involved in language processing in the human brain by analyzing these regions individually.

In this study, structural magnetic resonance allowed us to compute the structural connectivity (SC) between brain areas, and the application of the fingerprinting technique provided a method of comparing the similarity of interareal patterns of SC across the two species. For all key brain regions implicated in high-level linguistic processing, we chose an atlas used in previously published studies. The atlas was created by using a localizer, which was shown to robustly and reliably identify each of the key brain areas previously implicated in high-level linguistic processing in at least 80% of subjects individually and to be robust to changes in different stimulus modalities. We identified 11 high-level language processing regions in humans. Sixteen predefined homologous target regions were drawn for cross-species analysis, and the structural connection fingerprints of these homologous target regions were calculated. The results suggest that there are brain regions in macaques that display high similarity of structural coupling patterns relative to human high-level language processing regions. This is of great significance for understanding the functional specialization of human brain regions and the origin of language functions.

## 2. Materials and Methods

### 2.1. Subjects, Data Acquisition, and Preprocessing

The human data were collected from 22 healthy volunteers (aged 22–35 years) by means of the minimally preprocessed datasets provided by the Human Connectome Project (HCP) [22]. Data from the HCP dataset were acquired on a Siemens Skyra 3 T scanner at Washington University in St. Louis. The scanner was equipped with a customized body transmitter coil with a 56 cm bore size [22]. Structural MR images and diffusion MR images were used for the main analysis. High-resolution T1-weighted MR images were collected through the entirety of the brain with TR = 2400 ms and TE = 2.14 ms, and a voxel resolution = 0.7 × 0.7 × 0.7 mm. Diffusion-weighted images were acquired using echo-planar imaging (111 slices, TR = 5520 ms, TE = 89.5 ms, voxel resolution = 1.25 × 1.25 × 1.25 mm, no gap, acceleration factor = 3).

Macaque MRI data were obtained from the University of California, Davis (UC-Davis) via the PRIMatE Data Exchange (PRIME-DE) [23], including data for 19 rhesus monkeys aged between 18.5 and 22.5 years and weighing between 7.28 and 14.95 kg. All monkey MRI data were acquired in a Siemens Skyra 3T scanner with a 4-channel clamshell coil. The diffusion images were acquired with the following parameters: TR = 6400 ms, TE = 115 ms, voxel resolution = 1.4 × 1.4 × 1.4 mm, and slice gap = 1.4 mm. T1-weighted images were acquired with TR = 2500 ms, TE = 3.65 ms, voxel resolution = 0.3 × 0.3 × 0.3 mm, and flip angle = 7°.

All downloaded HCP data were preprocessed by the minimal preprocessing pipeline, the details of which have been described previously. The macaque diffusion MR images were processed by FSL6.0.5 software. The preprocessing steps included (1) the checking of data quality, including image parameters, head movement, artifacts, etc.; (2) eddy-current distortion and head motion correction using FSL’s Diffusion Toolbox; (3) the extraction of all macaques’ non-diffusion-weighted images (b = 0 s/mm^2^ from diffusion images and the removal of nonbrain tissue for all macaques and obtaining skull-stripped masks); and (4) tensor computation using FSL’s dtifit, followed by voxelwise crossing-fiber model-fitting of diffusion orientations using FSL’s BEDPOSTX (3 fibers per voxel; [24]). The extraction effect from using the FSL command “bet” on macaque data is relatively poor. Therefore, we did not use “bet” for the extraction, but instead used a ResTLU-Net model [25] to complete the extraction. Wang et al. have verified that the data processed by this model are reliable.

### 2.2. Definition of Regions of Interest (ROIs)

We determined language-related ROIs by combining the Human Brainnetome Atlas (BN Atlas) [26] with the language ROIs (allParcels_language_SN220). AllParcels_language_SN220 was determined by using Language localizer, which was developed to quickly and reliably identify brain regions previously implicated in linguistic processing [4,5]. These ROIs included six parcels: two regions in the left temporal lobe (LAntTemp and LPostTemp), two in the left inferior frontal gyrus (LIFGorb, LIFG), one in the left middle frontal gyrus (LMFG), and one extending into the angular gyrus (LAngG). The parcels are available for download from https://evlab.mit.edu/funcloc (accessed on 9 April 2024). Here, we selected brain regions with high overlap as ROIs by calculating the cross-combination between the BN Atlas and allParcels_language_SN220. Several ROIs were discarded because few voxels were mapped therein. Finally, we selected the following areas from the BN Atlas: Area 44, Area 45, angular gyrus (AG), Area 41/42, Caudal Area 22, the middle temporal gyrus, and the posterior superior temporal sulcus (Psts). In the final selected ROIs, LIFGrob and LIFG roughly correspond to Area 44 and Area 45 in the BN Atlas, LAngG corresponds to PGa and PGp, and LPostTemp contains Wernicke’s area in the posterior STG. The MTG has been parcellated into four subregions [27,28], and the Psts have been parcellated into two subregions [29], so we chose the subregion designations for subsequent analysis. After registering the BN atlas to the diffusion space for each subject, corresponding ROIs were extracted from the BN atlas.

For macaques, the D99 macaque atlas [30] was selected to study the relationships between macaque brain areas and human ROIs. Based on the anatomical position of the human ROIs, as many macaque brain regions as possible were expanded. Finally, the ROIs of the monkey brain were determined: all parcels in the lateral prefrontal cortex, agranular frontal cortex, inferior parietal lobule, and temporal lobe. The D99 atlas was registered to the diffusion space for each individual, and ROIs were extracted for subsequent research.

### 2.3. Probabilistic Tractography

To reduce the bias of tracking results caused by registering ROIs between the diffusion space and the template space, we registered the atlas to the subjects’ diffusion images and extracted these ROIs from the atlas based on the individual diffusion space. First, the subject’s non-diffusion-weighted image (b = 0 s/mm^2^) was registered to skull-stripped T1-weighted images from each participant using FSL’s FLIRT, and the parameter transforming an image in structural space into diffusion space after reversing was obtained. Second, the T1-weighted images were transformed to the Montreal Neurological Institute’s 152-brain template (MNI152) using FSL’s FLIRT and FNIRT, and parameters transforming an image in standard space into structural space were obtained after calculation. Finally, a transformation was performed to transform the BN atlas into diffusion space for each subject. The same procedures were performed on the D99 macaque atlas.

Probabilistic fiber tracking was performed in diffusion space using the FSL package. Voxelwise estimates of the fiber orientation distribution were calculated using Bedpostx. Drawing on these distributions, we estimated the fiber tracts between each voxel in each small cortical area and every voxel of the whole brain. This approach drew a sample from each fiber orientation distribution at the current voxel and chose the sample closest to the orientation identified in the previous step. The number of traces between each voxel in the seed area and target area is thought to be their connection probability. Five thousand streamline fibers per voxel were sampled for human probabilistic tractography, and 50,000 streamline fibers per voxel were sampled for macaque probabilistic tractography. The other parameters were set to their default values.

### 2.4. Comparison of Structural Connectivity between Macaque and Human Areas

A formal comparison between human and macaque brain area similarity was performed by calculating the cosine similarity (CS) and Manhattan distance (MD) of connectivity fingerprints. Connectivity fingerprints were proposed by Passingham and colleagues [31], who reported that the set of connections associated with each area is distinct from those of other brain areas. Currently, people use this method as a way to summarize the important connections of a single cortical area with a selected set of other areas, and it could also be used for data from different modalities. We also measured the differences in anatomical features between humans and macaques. The connectivity fingerprints are composed of seed areas and target areas. Seed areas are the brain areas the connections of which with other areas we plan to study, and target areas are known to be homologous between species. In our study, target regions were selected based on a published study. The strength of the connection between regions is measured by the number of traces; the more probabilistic fibers are traced, the stronger the connection. Since the absolute values of the probability counts vary widely between species, we used min–max normalization to control the result within a range between 0 and 1. The closer the result is to 1, the stronger the connection strength. The closer the cosine similarity value is to 1, the greater the similarity of the two connectivity fingerprints, and the greater the likelihood that the two brain regions are homologous. The smaller the value of the MD is, the greater the similarity of the two connectivity fingerprints, and the greater the possibility that the two brain regions are homologous. We are thus comparing patterns of connections with the target areas between humans and macaques, rather than absolute numbers.

## 3. Results

**Figure 1:**
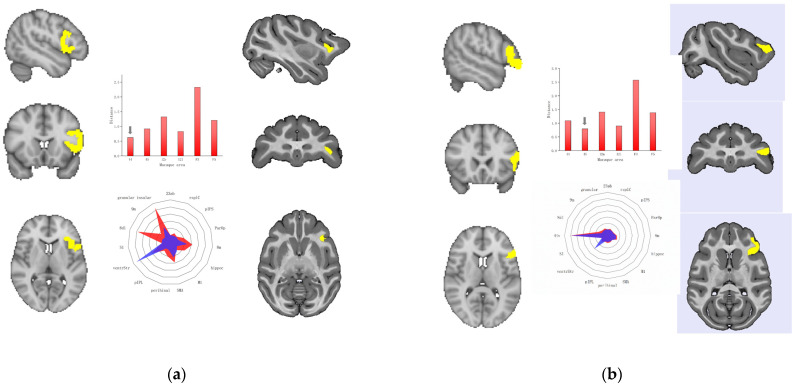
Comparison of structural connectivity between area 44 and area 45 of macaques and the Broca’s area of humans. The column on the left of each subfigure is a diagram of the spatial location of the human brain area. The column on the right is a diagram of the spatial location of the macaque brain area. In the middle column, the graph at the top is the Manhattan distance measure between the human fingerprint and each of the macaque connectivity fingerprints. The bottom fingerprint image shows the connections between human and macaque brain regions. (**a**) Comparison between human A44 and macaque area 44. (**b**) Comparison between human A45 and macaque area 45.

Whole-brain seed regions were identified on the basis of two principles: aligning allParcels_language_SN220 with the BN Atlas in standard space and extracting the corresponding parcellation from the BN Atlas; subsequently, the seed regions included in the language behavioral domain were retained. The finalized areas are as follows: area 44 (A44) and area 45 (A45) on the inferior frontal gyrus (IFG), angular gyrus (AG) on the inferior parietal lobule (IPL), rostroposterior superior temporal sulcus (rpSTS) and caudoposterior superior temporal sulcus (cpSTS) on the posterior superior temporal sulcus (pSTS), area 41/42 (A41/42) and caudal area 22 (A22c) on the superior temporal gyrus (STG), caudal area 21 (A21c), rostral area 21 (A21r), dorsolateral area 37 (A37dl), and anterior superior temporal sulcus (aSTS) on the middle temporal gyrus (MTG). Figure 1 shows the locations of these selected brain regions.

Whole-brain structural connectivity (SC) was calculated using species-specific seed regions for humans and macaques. For comparisons of interareal SC patterns between humans and macaques, we computed the SC of these seed regions with sixteen homologous regions [32]: the SMA, 44v, 8m, M1, S1, ParOp, pIPS, pIPL, 23ab, rsplC, perirhinal, ventrStr, hippoc, 9m, 8dl, and granular insula regions. Table 1 shows the corresponding labels of these sixteen regions in the BN Atlas and D99 Atlas. All experimental results were calculated by bringing brain regions of both species into a common coordinate system constructed from homologous brain regions.

A44 and A45 are subregions of Broca’s area, located in the posterior half of the left inferior frontal gyrus. In view of the existing comparative studies on human and monkey Broca regions, we investigated the lateral prefrontal cortex and agranular frontal cortex of macaques. We characterized structural connectivity for every brain area by calculating the number of white-matter fibers. We performed normalization because the absolute number of white-matter fibers differed significantly across species. To compare the SC fingerprints between humans and macaques, we created SC fingerprints for the Broca’s areas and selected brain areas of the monkey cortex based on 16 target ROIs. Then, we computed cosine similarities (CSs) and Manhattan distances (MDs) for pairs of macaque fingerprints and Broca’s areas. The results calculated by these two methods consistently showed that A44 of humans and area 44 of macaques had the highest similarity (CS = 0.98, MD = 0.62), and A45 of humans and area 45 of macaques had the highest similarity (CS = 0.97, MD = 0.79), among all preselected regions in macaques. We drew SC fingerprints for the Broca’s areas and their homologous areas, as shown in Figure 1a,b.

**Figure 2:**
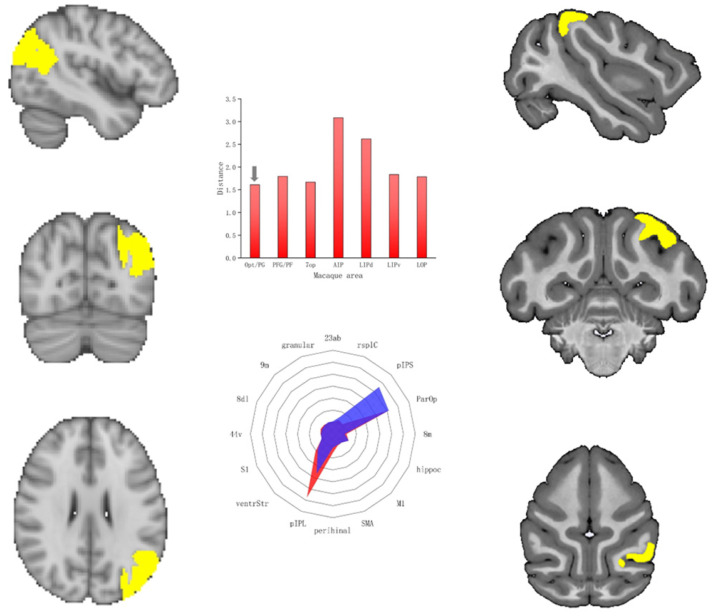
Comparison of structural connectivity between the angular gyrus of humans and the Opt/PG of macaques.

The angular gyrus is located in the posterior apex of the human inferior parietal lobe (IPL), at its interface with the temporal and occipital lobes. We used the brain regions of the inferior parietal lobule of macaques as candidate homologous brain regions in view of the existing research. We also computed CS and MD for SC fingerprints of the angular gyrus and macaque brain areas. Among these areas, the Opt/PG, located on the inferior parietal lobule (IPL) in macaques, displayed the greatest degree of cosine similarity (CS = 0.72, MD = 1.61). Figure 2 shows their SC fingerprints. In addition, we compared the structural connection characteristics of the angular gyrus of humans and the Opt/PG of macaques. The angular gyrus exhibited relatively high tractographic connectivity with the lateral superior occipital gyrus, caudal area 40, and nucleus accumbens. In contrast, it exhibited relatively weak connections with area 2, the dorsal granular insula, and the caudal area 24. Opt/PG showed strong connections with the PEa, parietal operculum, lateral intraparietal area, and ventral subdivision, and weak connections with the dorsomedial prefrontal area, dorsolateral prefrontal area, and 9 m.

**Figure 3:**
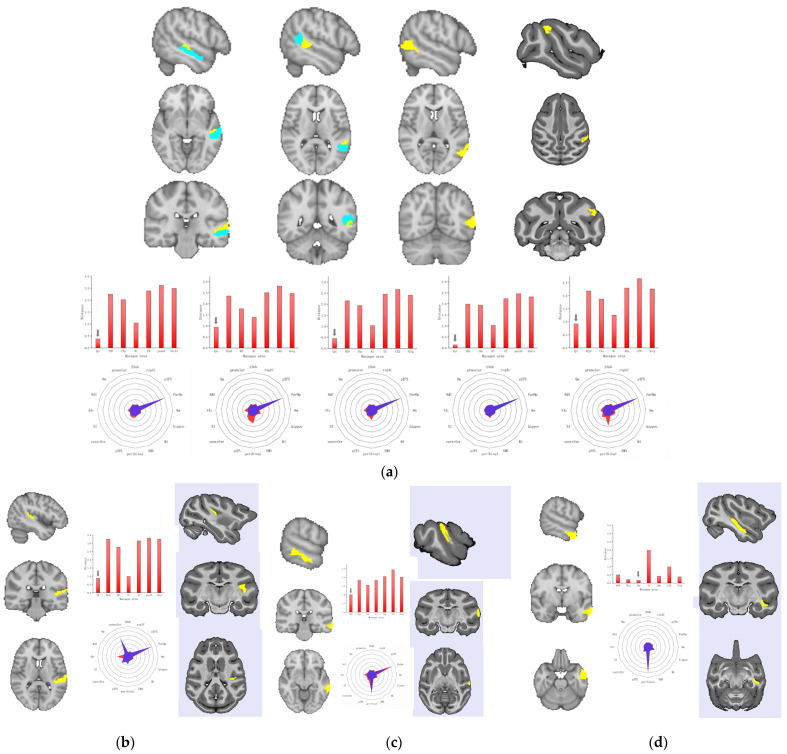
Comparison of structural connectivity between brain regions in the temporal lobe of humans and corresponding brain areas of macaques. In the D99 atlas, the temporal cortex consists of seven parts: the medial temporal lobe, inferotemporal temporal cortex (area TE), temporal pole cortex (TG area), auditory cortex, superior temporal gyrus, superior temporal sulcus (STS), and insular cortex. In the Manhattan-distance bar chart, each column shows the corresponding area of the minimum distance of each part. (**a**) Comparison between A22c, aSTS, rpSTS, cpSTS, and A37dl of humans and Tpt of monkeys. (**b**) Comparison between A41/42 of humans and CM of monkeys. (**c**) Comparison between A21c of humans and CPB of monkeys. (**d**) Comparison between A21r of humans and IPa of monkeys.

In addition, other brain areas are distributed in the human temporal lobe. Accordingly, we performed a blind search across the temporal cortical territory of macaques to test whether there were any areas in the macaque brain that showed high similarity to human areas. We used the same method to calculate the CS and MD of the SC fingerprints for human brain regions and all temporal lobe brain regions of monkeys. The results showed that A22c, rpSTS, cpSTS, aSTS, and A37dl in the human brain all showed the highest similarity with the area Tpt in the macaque brain (A22c: CS = 0.991, MD = 0.379; rpSTS: CS = 0.983, MD = 0.445; cpSTS: CS = 0.997, MD = 0.142; aSTS: CS = 0.936, MD = 0.943; A37dl: CS = 0.930, MD = 0.923). Figure 3a shows their SC fingerprints. The CM area in macaques was most similar to that in A41/42 (CS = 0.952, MD = 0.881). The CPB area in macaques was most similar to that in A21c (CS = 0.952, MD = 1.003). A21r in humans displayed high similarity with the IPa in macaques (CS = 0.998, MD = 0.171). The SC fingerprints correspond to Figure 3a–d.

## 4. Discussion

Language is a complex cognitive function that is considered unique to human beings. In fact, the generation and understanding of language is the result of the joint action of the frontal, temporal, and parietal lobes, and these brain structures also exist in the monkey brain. However, few studies have focused on the relationships between species in these areas. In this study, we focused only on the structural characteristics of brain regions, and we compared the SC patterns among species using diffusion tensor imaging (DTI) data. We searched throughout the macaque frontal, parietal, and temporal cortices for areas with SC connectivity highly similar to that found in human language areas. Our results demonstrate that these brain regions involved in human brain language-function can be associated with brain regions with similar structural patterns found in the monkey brain.

In this study, we found that area 44 and area 45, which are located in the lateral prefrontal cortex of the monkey brain, have the highest structural-connection similarity with A44 and A45, two subregions of the human Broca region. This result is consistent with that of a previous report [16,33]. It has been confirmed by combining quantitative architectonic analysis of the cortical areas with electrophysiological recording of neuron activity and electrical intracortical microstimulation that the homolog of cytoarchitectonic A44 (Broca’s area) is the monkey area 44 [32]; the Broca region of the human brain compares with the homolog region in the brain of nonhuman primates from the perspective of cell structure and receptor structure. In addition, the brain connectivity in the human Broca’s area exhibits similarities with those of its arguable homologs in macaques [12,16].

Previous studies have divided the human angular gyrus into two subregions based on cell structure and receptor structure: the caudal PGp and rostral PGa. Architectonic studies revealed the existence of six architectonically distinct areas within macaque area 7, and further connectivity and functional imaging studies have supported the hypothesis that Opt and PG may constitute the homologs of the human areas PGp and PGa, respectively [12]. Some studies have also comprehensively analyzed PGp and PGa in the human brain and their corresponding structures in the rhesus monkey brain from the view of the cyto-, myelo- and receptor-architecture. In this study, the angular gyrus corresponded to the PGp and PGa. The brain region that displayed the highest level of structural similarity to the angular gyrus corresponded to the Opt/PG in macaques. Therefore, our results supported the homology of the angular gyrus from the perspective of structural connections.

The human temporal lobe is the brain area most involved in advanced language processing. We found the brain region with the highest level of connection similarity relative to the human brain region within the temporal lobe of the macaque. Our results suggest that area 41/42 on the STG of humans corresponds to the caudomedial field on the lateral sulcus of macaques, and caudal area 21 on the middle temporal gyrus of humans corresponds to the CPB of macaques. In addition, caudal area 22 on the STG, dorsolateral area 37 on the MTG, and the anterior superior temporal sulcus and posterior superior temporal sulcus all displayed high levels of similarity with the Tpt of macaques. Rostral area 21 on the MTG showed a high level of similarity with the fundus area in the superior temporal sulcus. In general, these brain regions with high structural similarity are located in the lateral sulcus, temporal plane, and part of the superior temporal sulcus of the monkey brain. Indeed, existing studies generally hold that the human temporal lobe is expanded compared with those of nonhuman primates. Based on the comparison of gyral and sulcal landmarks in humans and macaques, the superior, middle, and inferior temporal cortices all displayed evident expansions [34]. Compared with that of macaques, the primary auditory cortex of humans covers a smaller proportion of the cortical surface area in the superior temporal plane and is located further back [35]. The relative location of the visual motion area MT has also been modified: it has been displayed posteriorly and inferiorly in humans, as compared to macaques. Moreover, the cortical areas intervening between the auditory core and MT have expanded, and expansion of the anterior temporal cortex has occurred [36]. In this case, structural connections provide important insights for comparing the relationships between human and monkey brain regions. Structural connectivity reflects the anatomical organization of the brain by means of its fiber tracts. It has been demonstrated to be effective in predicting anatomical connections that determine the flow and nature of information in the cortex. In the temporal lobe of the monkey brain, we found brain regions with high connectivity-similarity with human language-processing regions. This indicates that, although functional specialization of brain regions has occurred during the evolutionary process, they may have a common structural precursor. This research provides an important step toward exploring the language function of humans. However, there are several important questions to be studied: In the process of evolution, how did the differences in the brain regions of humans and monkeys arise? How does structure affect the specialization of brain function? Our future research will continue to explore these issues.

## Figures and Tables

**Table 1:** Homologous human and macaque target regions.

Brain Area	BN_246_Name	D99_Name
44v	IFG_6_6	44
SMA	SFG_7_5	F3
8m	SFG_7_3	8Bm
M1	PoG_4_1	F1_(4)
S1	PoG_4_3	3a/b
ParOp	IPL_6_4	7op
pIPS	SPL_5_3	5_(PEa)
pIPL	LOcG_2_2	LIPv
23ab	CG_7_6	23b
rsplC	CG_7_4	30
perirhinal	ITG_7_3	IPa
ventrStr	BG_6_3	Striatum
hippoc	Hipp_2_2	CA3
9m	SFG_7_6	9m
8dl	SFG_7_2	8Bd
granular insula	INS_6_5	lg

## Data Availability

The data presented in this study are available. The human data are accessible at https://www.humanconnectome.org/study/hcp-young-adult/ (accessed on 9 April 2024). The macaque data are accessible at http://fcon_1000.projects.nitrc.org/indi/PRIMEdownloads.html (accessed on 9 April 2024).
